# Clinical characteristics of 683 children and adolescents, aged 0–18 years, newly diagnosed with type 1 diabetes mellitus in Henan Province: a single-center study

**DOI:** 10.1186/s12887-023-03847-z

**Published:** 2023-01-23

**Authors:** Ai Huang, Qiong Chen, Wei Yang, Yan Cui, Qingzhi Wang, Haiyan Wei

**Affiliations:** 1 Department of Endocrinology, Children’s Hospital Affiliated to Zhengzhou University, Henan Children’s Hospital, Zhengzhou Children’s Hospital, 33 Longhu Outer Ring East Road, Zhengzhou, Henan 450018 People’s Republic of China; 2 Vanke School of Public Health, Tsinghua University, Beijing, China

**Keywords:** Glycated hemoglobin A, Diabetes mellitus, Hyperglycemia, Diabetic ketoacidosis, Blood glucose, C-peptide

## Abstract

**Background:**

Type 1 diabetes mellitus (T1DM) is a common chronic systemic disease that threatens the health of children worldwide. Diabetic ketoacidosis (DKA) is the most severe acute complication of diabetes and can lead to death. This study aimed to explore the epidemiological features, clinical manifestations, and risk factors for DKA in children and adolescents newly diagnosed with T1DM in the Department of Endocrinology of the Children’s Hospital of Henan Province.

**Methods:**

Medical records of 683 children and adolescents newly diagnosed with T1DM in our center from March 2014 to November 2021 were retrospectively analyzed. The data included the general condition, laboratory indexes, and clinical symptoms. The patients were divided into three groups according to age: Group I, 0–3 years; Group II, 4–9 years; and Group III, 10–18 years.

**Results:**

The incidence of DKA was 62.96% and was highest in Group I. Group I had the lowest C-peptide and hemoglobin A1c, but the highest blood glucose at first diagnosis, and 25-hydroxyvitamin D3 levels, hospitalization lengths, and medical costs. 25.5% of the children were delayed in diagnosis. Logistic regression analysis showed that elevated HbA1c levels and hyperglycemia were independent risk factors for DKA. On the other hand, C-peptide and 25- hydroxyvitamin D were protective factors for DKA.

**Conclusions:**

The incidence of DKA among children and adolescents in the Henan Province is very high. Moreover, DKA can be easily delayed in diagnosis. Newly diagnosed infants with T1DM are more likely to present with DKA, suffer more severe metabolic disorders, endure longer hospital stays, and accrue higher medical costs.

## Background

Type 1 diabetes mellitus (T1DM) is one of the most common chronic diseases and major health threats in children. The reported incidence of T1DM ranges from 0.1/100,000 per year in China and Venezuela to 52.2/100,000 per year in Finland [1, 2]. In the past few decades, the global annual incidence of T1DM in children has been increasing at a rate of 2–3% per year [3]. The incidence of T1DM among Chinese children is relatively low; however, the total number of T1DM cases is high due to the large population of China. This places a heavy burden on families and society. Research shows that diabetes patients in China reached 1.14 million, and the related medical expenses exceed 60 billion US dollars; About 7% ~ 12% are T1DM. Most patients with T1DM are children and adolescents. There are about 47,000 T1DM patients under the age of 20 years old in China, with about 6000 new cases every year [4].

Of the cases of childhood diabetes, T1DM accounted for approximately 90%, type 2 diabetes and other special types accounted for approximately 10%. T1DM, also known as insulin-dependent diabetes, is a chronic autoimmune disease caused by T-cell-mediated islet beta cell destruction and absolute insulin deficiency. The pathogenesis of T1DM is still unclear, and it is believed to be the result of the combined action of genetic, environmental, immune, and other factors [5].

Diabetic ketoacidosis (DKA) is an acute complication characterized by hyperglycemia, acidosis, and ketosis. It presents with dehydration, vomiting, abdominal pain, disturbance of consciousness, and rapid deep respiration. DKA is the leading cause of death in children with T1DM because of hypokalemia, deep vein thrombosis, and cerebral edema [6, 7]. The International Society of Pediatric and Adolescent Diabetes (ISPAD) pointed out that DKA is the most common cause of death in children with diabetes, with the highest mortality rate among newly diagnosed patients with T1DM [8]. Nonetheless, DKA is preventable; therefore, awareness among primary physicians and the general public should be enhanced to reduce the incidence of DKA.

Large sample epidemiological data and clinical studies on DKA in children are lacking in China. In this study, we retrospectively analyzed the clinical data of 683 children with newly diagnosed T1DM in our center. Our center is a class-A third-grade children’s hospital in Henan Province (the central region of China), and it hosts patients from all over the province. This study aims to describe characteristics of new-onset childhood T1DM in China, and improve early diagnosis and treatment.

## Methods

This study included children and adolescents who were newly diagnosed with diabetes mellitus in the Department of Endocrinology of Children’s Hospital Affiliated to Zhengzhou University in Henan Province in the last 8 years, from 2014 to 2021. The patients were divided into three groups according to age: Group I, 0–3 years; Group II, 4–9 years; and Group III, 10–18 years.

The study was approved by the research and ethical committees of the hospital.

### Inclusion and exclusion criteria

Patients who met the World Health Organization (WHO) diagnostic criteria of T1DM were included in the study. According to the WHO in 2021 [9], T1DM can be diagnosed by the following characteristics: an acute onset, a thin body type; often suffering from ketosis or ketoacidosis; significantly low fasting or postprandial C peptide; the presence of one or more islet autoantibodies and being on insulin dependent therapy. Patients with negative autoantibodies are considered to have idiopathic type 1 diabetes. All the children and adolescents in this study were correctly diagnosed according to the WHO diagnosis criteria above. Patients with type 2 diabetes, secondary diabetes, or special type diabetes were excluded.

### Observation index

The following data were collected:

1. General information—age, sex, birth history, eating pattern, occupation of parents, family history, and onset season.
2. Laboratory tests—hemoglobin A1c (HbA1c), C-peptide, blood glucose, pH, residual alkali, bicarbonate, diabetes autoantibodies: islet cell autoantibody (ICA), insulin autoantibody (IAA), glutamic acid decarboxylase antibody (GADA), and 25-hydroxyvitamin D (25OHD) level. IA-2A, ZnT8a antibodies were not routinely measured.
3. Clinical data—symptoms at onset, such as polydipsia, polyuria, polyphagia, and weight loss; DKA symptoms such as deep breathing, abdominal pain, nausea, and vomiting; delayed diagnosis refer to a clinical encounter when the child was discharged from care with the incorrect diagnosis before diabetes diagnosis, and brought to the hospital later; hospital length of stay, and cost of hospitalization.

### Diagnostic criteria and classification of DKA

According to ISPAD Clinical Practice Consensus Guidelines 2014 [10], Diagnostic criteria of diabetes ketoacidosis: blood glucose > 11 mmol / L, ketonemia and ketonuria, blood gas analysis pH < 7.3, bicarbonate (HCO3 -) < 15 mmol /L. DKA was categorized into three degrees: mild, arterial pH decreased to between 7.2 and 7.3 and serum bicarbonate level decreased to 10–15 mEq/L; moderate, arterial pH of 7.1–7.2 and a bicarbonate level of 5–10 mEq/L; severe, arterial pH below 7.1 and a bicarbonate level below 5 mEq/L.

### Statistical methods

All the results were processed using SPSS software, version 23.0 (IBM Corp., Armonk, NY, USA). Normally distributed measurement data are expressed as means and standard deviations. Categorical data are presented as percentages. Non-normally distributed data are presented as medians and quartiles. The *t*-test was used to compare differences in numerical variables between two groups if they conform to the normal distribution, if not, we used the rank sum test (Wilcoxon signed rank test). Univariate analysis of variance was used to compare the mean difference among multiple numerical groups. The Chi-square test was used to compare the rates between groups. After univariate analysis, the significant group was entered into a multivariate logistic regression analysis for statistical operation. Bilateral *P* < 0.05 were considered statistically significant.

## Results

### Distribution of age, gender, and onset season

**Fig. 1:**
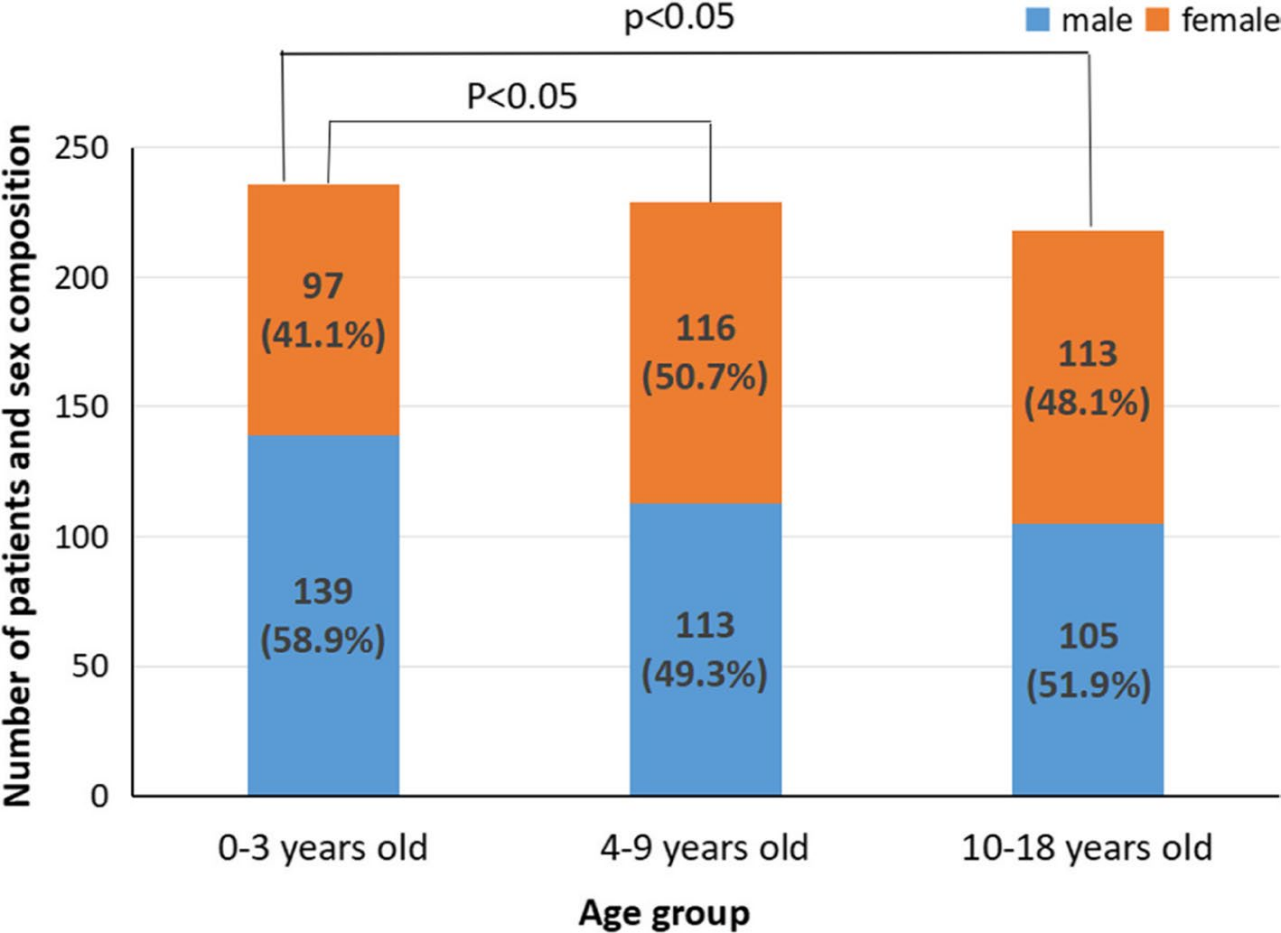
Age and sex distribution at T1DM onset. T1DM, type 1 diabetes mellitus

A total of 683 children with newly diagnosed T1DM in the last 8 years were included in the study, 357 were males and 326 were females (male: female ratio, 1.1:1). There was no significant difference in sex ratio (*P* = 0.236). The average age of onset was 7.91 ± 4.85 years (range, 0.2–18 years). There were 117 males (61.90%) and 72 females (38.10%) in Group I, 124 males (48.44%) and 132 females (51.56%) in Group II, and 116 males (48.74%) and 122 females (51.26%) in Group III. The age and sex distributions are shown in Fig. 1.

**Fig. 2:**
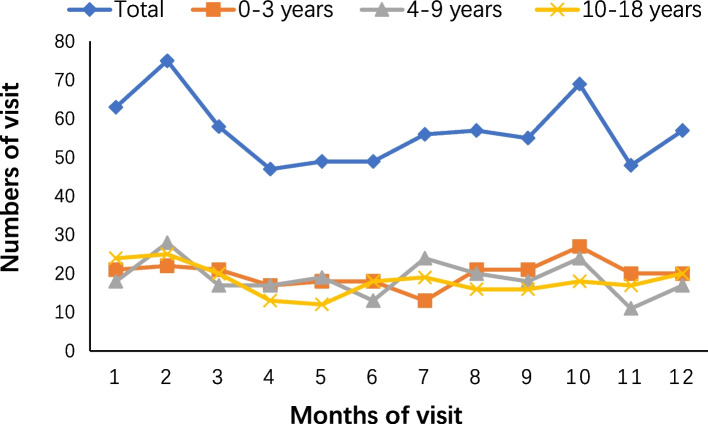
Distribution of the first medical visits by month before recognition of diabetes

The distribution of the first medical visits in each month is shown in Fig. 2. Henan Province, where we live, is a province in Central China. It has four distinct seasons. It is spring from the middle of February to the middle of May every year. It is summer from the middle of May to the middle of August. Autumn is from the middle of August to the middle of November. Winter is from the middle of November to the middle of February of the next year. There were 154 cases (22.55%) in spring, 169 cases (24.74%) in summer, 170 cases (24.89%) in autumn, and 190 cases (27.82%) in winter. Winter is the season with the highest incidence of T1DM.

### Clinical signs and symptoms

**Fig. 3:**
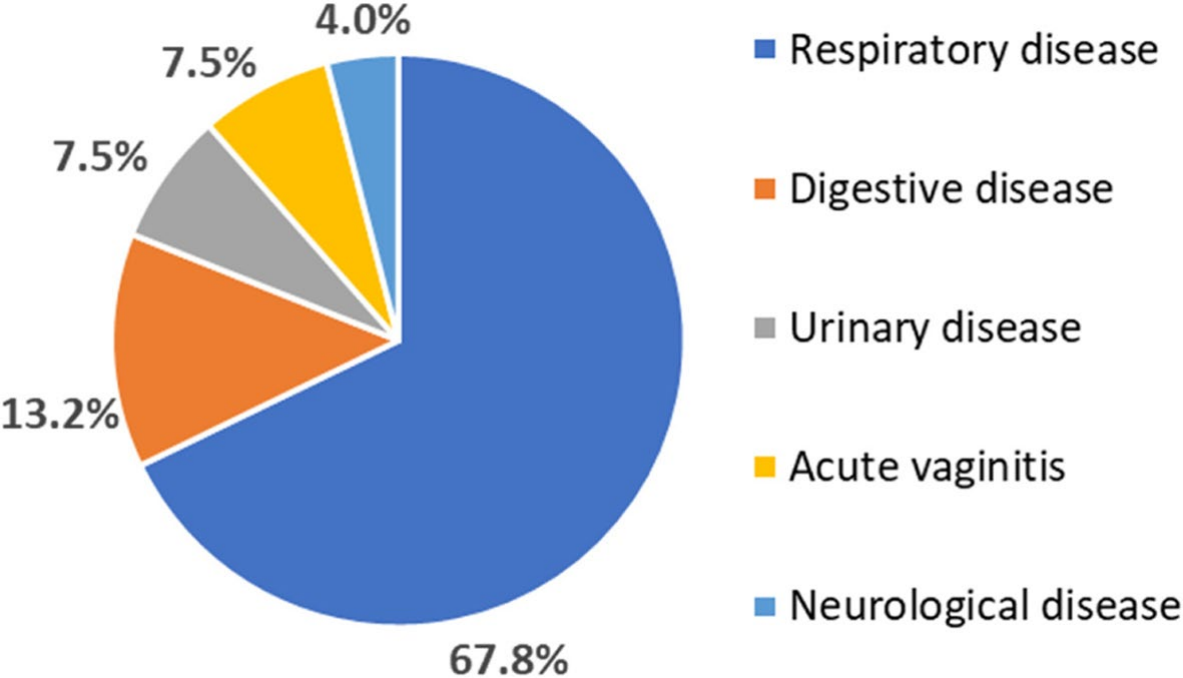
Analysis of delayed diagnosis of DKA in newly diagnosed diabetes patients (*n* = 174). DKA, diabetic ketoacidosis

A total of 458 patients (62.96%) presented with DKA at onset. Mild DKA accounted for 43.46% of these cases and moderate and severe DKA accounted for 56.54%. A total of 225 patients (32.94%) did not have DKA at first diagnosis. At initial diagnosis, 541 patients (79.21%) had polydipsia, 494 patients (72.33%) had polyuria, only 138 patients (20.2%) had polyphagia, and 225 patients (32.94%) showed weight loss. A total of 130 patients (19.03%) had a family history of diabetes mellitus. The positive rates of three autoantibodies (GADA, IAA, and ICA) in our research were 50.6%, 24.0%, and 4.1%, respectively, with *P* < 0.05. A total of 174 cases were delayed in diagnosis at the time of onset. Among the patients with a delayed diagnosis, 67.8% were initially thought to have respiratory diseases, 13.2% digestive diseases, 7.5% urinary tract diseases, 7.5% vaginitis, and 4% a central nervous system disorder (Fig. 3).

### Difference analysis of each age group

We used the Chi-squared test to compare the difference of each index among the three groups. There were no statistical differences in the onset season, family history, DKA degree, breast milk, and disease course among the three age groups. There were statistically differences in sex, the incidence of DKA, C-peptide, glycosylated hemoglobin at first diagnosis, blood glucose at first diagnosis, 25OHD level, hospital length of stay, hospitalization costs, and the incidence of polydipsia, polyuria, weight loss, and complicated systemic diseases among the three groups (Table 1).

**Table 1:** Age difference analysis of each index

	0–3 years (*n* = 236)	4–9 years (*n* = 229)	10–18 years (*n* = 218)	*P*
**Sex (male)**	139 (58.9%)^a^	113 (49.3%)^b^	105 (48.1%)^b^	0.04*
**Onset season**				
Spring	49 (20.76%)	50 (21.83%)	55 (25.23%)	0.318
Summer	54 (22.88%)	65 (28.38%)	50 (22.94%)	
Autumn	70 (29.66%)	53 (23.14%)	47 (21.56%)	
Winter	63 (26.69%)	61 (26.64%)	66 (30.28%)	
**Family history positivity**	48 (20.34%)	41 (17.90%)	41 (18.81%)	0.795
**DKA positivity**	167 (70.76%)^a^	134 (58.52%)^b^	129 (59.17%)^b^	0.009*
**DKA degree**				
Mild	64 (38.32%)	64 (47.76%)	60 (46.51%)	0.195
Moderate and severe	103 (61.68%)	70 (52.24%)	69 (53.49%)	
**Term infant**	223 (94.5%)^a^	194 (84.7%)^b^	127 (58.3%)^c^	< 0.001*
**Breast milk before 6 months**	190 (80.5%)	176 (76.9%)	163 (74.8%)	0.332
**HbA1c (%)**	11.19 (2.44)^a^	12.36 (2.47)^b^	12.24 (3.09)^b^	< 0.001*
**C-peptide** (nmol/L)	0.31 (0.14,0.42)^a^	0.52 (0.24,0.46)^b^	0.74 (0.28,0.75)^b^	< 0.001*
**Blood glucose** (mmol/L)	23.43 (6.61)^a^	22.94 (7.37)^a^	20.36 (7.21)^b^	< 0.001*
**25OHD (ng/ml)**	20.73 (9.54)^a^	17.79 (7.19)^b^	17.08 (6.55)^b^	< 0.001*
**Hospital length of stay (days)**	15.88 (6.55)^a^	14.32 (6.07)^b^	11.60 (5.45)^c^	< 0.001*
**Disease course (days)**	5 (0,15.5)	5 (0,15)	4.5 (0,12)	0.922
**Cost (China Yuan)**	7481.52 (6166.42,10,495.78)^a^	6819.28 (5304,8492.66)^b^	6226.4 (4298.39,8570.15)^b^	< 0.001*
**Polydipsia**	206 (87.29%)^a^	192 (83.84%)^a^	155 (71.10%)^b^	0.002*
**Polyuria**	191 (80.93%)^a^	179 (78.17%)^a^	124 (56.88%)^b^	< 0.001*
**Weight loss**	56 (23.73%)^a^	93 (40.61%)^b^	76 (34.86%)^b^	< 0.001*
**Delayed diagnosis**	83 (35.2%)^a^	50 (21.8%)^b^	41 (18.8%)^b^	< 0.001*
**ICA positivity**	2 (0.8%)^a^	12 (5.2%)^b^	14 (6.4%)^b^	< 0.001*
**IAA positivity**	42 (17.8%)^a^	26 (11.3%)^b^	96 (44%)^c^	< 0.001*
**GADA positivity**	140 (59.3%)^a^	138 (60.2%)^a^	68 (31.1%)^b^	< 0.001*

^a,b,c^ means Statistical difference between groups

* means *P* value< 0.05, statistically significant among the three groups

*25OHD* 25-hydroxyvitamin D, *HbA1c* Hemoglobin A1C, *DKA* Diabetic ketoacidosis, *ICA* Islet cell autoantibody, *IAA* Insulin autoantibody, *GADA* Glutamic acid decarboxylase antibody

There was a statistically significant difference in the sex ratio among the three groups, *P* < 0.05. The proportion of male children was highest in Group I; however, there was no statistical difference in this proportion between Groups II and III. The incidence of T1DM in male children was higher in the infant group.

Group I had the highest incidence of DKA (70.76%), *P* = 0.009. There was no statistically significant difference in the incidence of DKA between Groups II and III.

The lowest C-peptide and HbA1c levels at first diagnosis were found in Group I, *P* < 0.001; however, no statistical difference was found between Groups II and III.

The blood glucose level of Group I and II at first diagnosis was higher than Group III, *P* < 0.001; however, there was no statistical difference between the first two groups.

The 25OHD level in Group I was significantly higher than that in the other two groups, *P* < 0.001; however, there was no statistical difference between the other two groups.

There were differences in hospital length of stay among the three groups: Group I > Group II > Group III, *P* < 0.001. The hospitalization cost in Group I (7481.52 yuan) was significantly higher than that in the other two groups, *P* < 0.001; however, there was no statistical difference between Groups II and III.

The incidence of and polyuria in Group I and II was significantly higher than Group III, *P* = 0.02. The incidence of polydipsia and polyuria in Group I and II was significantly higher than Group III, *P* < 0.001. Groups II and III had a higher incidence of weight loss than Group I, *P* < 0.001.

Percentage of the delayed diagnosis in Group I was higher than that of the other two Groups, *P* < 0.001. The difference was statistically significant.

There were statistical differences among the three antibodies in each Group. The positive rate of ICA: Group II and Group III > Group I, *P* < 0.001. The positive rate of IAA: Group III > Group I > Group II, *P* < 0.001. The positive rate of GADA: Group I and Group II > Group III, *P* < 0.001.

### Analysis of influencing factors for DKA

#### Univariate analysis of DKA

We included age, sex, parental occupation, onset season, family history, HbA1c, C-peptide, blood glucose, 25OHD, and autoantibody in the univariate analysis of factors for DKA. There were no differences between DKA and non-DKA in sex, parental occupation, onset season, family history, and autoantibody. Compared with the non-DKA group, the DKA group showed younger age, higher HbA1c and blood glucose, lower C-peptide and vitamin D. Univariate analysis of DKA showed that age, C-peptide, and 25OHD were negatively correlated with DKA, while HbA1c and blood glucose were positively associated with DKA **(**Table 2**)**.

**Table 2:** Univariate analysis of DKA

	DKA (***n*** = 430)	Non DKA (***n*** = 253)	***P*** value
**Age (years)**			0.009*
0–3 years	167 (38.8%)	69 (27.3%)	
4–9 years	134 (31.1%)	95 (37.5%)	
10–18 years	129 (30%)	89 (35.2%)	
**Sex (male)**	220 (51.2%)	137 (31.9%)	0.45
**Father occupation**			0.664
Industrial and agricultural workers	201 (46.7%)	105 (41.5%)	
Service workers	22 (5.1%)	14 (5.5%)	
Civil servants and employees of enterprises and institutions	43 (10%)	22 (8.7%)	
Professional and technical staff	10 (2.3%)	8 (3.2%)	
Self-employment	72 (16.7%)	45 (17.8%)	
Other	82 (19.1%)	59 (23.3%)	
**Mother occupation**			0.717
Industrial and agricultural workers.	181 (42.1%)	96 (37.9%)	
Service workers	15 (3.5%)	13 (5.1%)	
Civil servants and employees of enterprises and institutions	45 (10.5%)	22 (8.7%)	
Professional and technical staff	6 (1.4%)	4 (1.6%)	
Self-employment	61 (14.2%)	40 (15.8%)	
Other	122 (28.4%)	78 (30.8%)	
**Onset season**			0.167
Spring	94 (21.9%)	60 (23.7%)	
Summer	98 (22.8%)	71 (27.4%)	
Autumn	118 (27.4%)	52 (20.5%)	
Winter	120 (27.9%)	70 (27.7%)	
**Family history positivity**	76 (17.7%)	54 (21.3%)	0.238
**HbA1c at onset (%)**	12.11 ± 2.66	11.59 ± 2.80	0.017*
**C-peptide at onset (nmol/L)**	0.28 (0.16,0.43)	0.42 (0.28,0.67)	< 0.001*
**Blood glucose at onset (mmol/L)**	23.43 ± 6.91	20.34 ± 7.21	< 0.001*
**25OHD (ng/mL)**	17.99 ± 7.72	19.57 ± 8.50	0.013*
**ICA positivity**	18 (4.1%)	10 (3.9%)	0.882
**IAA positivity**	105 (24.4%)	59 (23.3%)	0.746
**GADA positivity**	220 (51.2%)	126 (49.8%)	0.731

^*^means *P* value< 0.05, statistically significant between the two groups

*25OHD* 25-hydroxyvitamin D, *HbA1c* Hemoglobin A1C, *DKA* Diabetic ketoacidosis, *ICA* Islet cell autoantibody, *IAA* Insulin autoantibody, *GADA* Glutamic acid decarboxylase antibody

#### Multivariate logistic regression analysis of DKA

Further logistic regression analysis found that the independent risk factors for DKA included elevated HbA1c levels and elevated blood glucose levels. On the other hand, 25OHD and C-peptide were protective factors for DKA **(**Table 3**)**.

**Table 3:** Results of logistic regression analysis

	OR	SE	***P***	95%CI	
**Age (0–3 years as reference)**			0.091		
**4–9 years**	1.209	0.114	0.409	0.77	1.900
**10–18 years**	0.759	0.165	0.192	0.501	1.148
**HbA1c**	1.067	0.032	0.042	1.002	1.137
**C-peptide**	0.415	0.213	< 0.001	0.273	0.631
**Blood glucose**	1.055	0.012	< 0.001	1.030	1.081
**25OHD**	0.970	0.011	0.007	0.949	0.992

*OR* Odds ratio, *SE* Standard error, *25OHD* 25-hydroxyvitamin D, *HbA1c* Hemoglobin A1C, 95%CI 95% confidence interval.

## Discussion

There was no apparent peak age for newly diagnosed T1DM in the Henan Province. In Iran and China Hangzhou, the peak age of onset is between 5 and 9.9 years [11, 12], while in China Beijing, Japan, and Uzbekistan, it was in adolescence group [13–15]. Our study revealed that diabetes in children 0–3 years old accounted for 34.5%. This conclusion deserves our attention. According to Adeloye D [16], the incidence of T1DM in children aged 0–4 years is rising worldwide, and was 20.9 (7.8–34.1) per 100,000 child years in 2015. The incidence of T1DM is shifting towards younger age groups. According to the newest census data from National Bureau of Statistics of China (http://www.stats.gov.cn/tjsj/pcsj/), children aged 0–4 years account for 21.2% of all the children in Henan Province, children aged 5–9 years account for 28.5% and 10–19 years account for 50.3%. It suggests that the proportion of younger children with T1DM is higher than their proportion of the population. We analyzed the possible reasons for the high proportion of infant diabetes as follows: Our hospital is the largest a class-A third-grade specialized children’s hospital in Henan Province, with advanced professional technology. Thus, parents of young children tend to choose our hospital, while those of older children may choose other general hospitals.

Although there was no significant difference in sex ratio, our study suggested that the incidence of T1DM in male children was higher in Group I. According to National Bureau of statistics of China, among healthy children aged 0–4 in Henan Province, boys accounted for 52.06% and girls 47.94%. The reason for this phenomenon was not very clear and may be related to the gender composition differences and complex environmental factors.

Our research showed the incidence of T1DM peaked in winter. The seasonal pattern of T1DM onset is well known and has been confirmed repeatedly. In a study from Sweden, the incidence was higher from January to March and lowest from May to July [17]. It may be related to the high incidence of virus infections in this season.

The main symptoms before the hospital visit were polyuria (72.33%) and polydipsia (79.21%), followed by weight loss (32.94%) and polyphagia (20.2%). A study from Taiwan showed that the most common symptoms were polyuria (96%), polydipsia (92%), dry lips (81%), and weight loss (79%) [18]. In this study, it was found that polydipsia and polyuria were more common in infants and school-age children with T1DM, while weight loss was more common in adolescent children. This may be related to the strong self-consciousness of adolescent children and the difficulty in timely detection of symptoms.

Our study shows that delayed diagnosis occurs in 25.5% of newly diagnosed patients, and is especially commen in group I. DKA is diagnosed as respiratory disease simply because acidosis status can lead to deep breathing. In addition, such patients are usually prone to respiratory tract infections. The reason for gastrointestinal discomfort caused by DKA may be the stimulation of gastrointestinal mucosa by metabolites produced by acidosis; electrolyte disorder Metabolic acidosis leads to spasms of gastrointestinal smooth muscle and even paralytic intestinal obstruction.

Children could not accurately express the typical symptoms of diabetes, such as polydipsia and polyuria, and caregivers and doctors had insufficient knowledge of the disease; therefore, the diagnosis of DKA was easily delayed, especially in younger children.

One major finding is that 62.96% of the children presented with DKA in this study, moderate and severe DKA accounted for more than half of the DKA cases. The incidence of DKA varies in different countries and regions [14], with the lowest incidence reported in Denmark (14.7%) and the highest in Saudi Arabia (79.8%). Results of multi-center epidemiological investigations are lacking in China. The incidence of DKA in newly onset Children with T1DM of China Hangzhou was 50.1% [12]. The incidence rate of TIDM in China is low, but the incidence of DKA is particularly high. We suspect it may be related to the insufficient understanding of the disease by the general public and even pediatricians.

Many studies have shown that young age, race, residing in a rural area, and lack of health insurance are the main risk factors for DKA [19, 20]. In our research, age, blood glucose, HbA1c, C-peptide, and 25OHD were associated with DKA. However, we found that age was not an independent risk factor for DKA after multivariate logistic regression analysis. This suggested that age may be a confounding factor for DKA. The blood glucose and HbA1c level in the DKA group increased significantly, which suggested more serious metabolic disorder in the DKA group. C-peptide can reflect islet β Cell function was not affected by exogenous insulin and endogenous insulin antibody. Children with more serious islet β Cell function impairment at first diagnosis were more prone to DKA. According to Agnieszka Szypowska [21], the level of C-peptide in children with DKA is lower than those without DKA, which is consistent with our study.

The positive rates of autoantibodies for GADA, IAA, and ICA in our research was 50.6%, 24%, and 4.1%, respectively. In a multicenter survey in Chinese children, the proportions of children aged 0–14 years were positive for GADA, IAA, and ICA, were 29.4%, 15.2%, and 11.8% [22]. Studies from Brazil and Australia show that the antibody positive rate is higher than that of Chinese children. This may be related to ethnic differences and age distribution. Although the appearance of diabetes autoantibodies is a high-risk factor for children with T1DM, in our study, diabetes antibodies didn’t predict the occurrence of ketoacidosis.

In our study, the 0–3-year-old group had the highest incidence of DKA. This may be related to the fact that the child is too young to accurately express symptoms. The 0–3-year-old group had the highest blood glucose levels and the lowest C-peptide levels. This reflected that the islet β cell function damage and metabolic disorders were more serious at onset in the young age group. Interestingly, the 0–3-year-old group had the lowest HbA1c levels. This also prompted the more rapid and severe damage of insulin β cells caused by immune injury, leading to a more serious metabolic disorder. This is consistent with the results of a study conducted in Taiwan [18]. Hospital length of stay and hospitalization costs were highest in the youngest age group. This was possibly related to the high morbidity of DKA and the complications with other systemic diseases in children in the young age group.

According to our findings, vitamin D deficiency is widely prevalent in children with T1DM in the Henan Province. The serum 25OHD levels among the children in the 0–3-year-old group were significantly higher than those of the children in the other two groups. Further, there was no statistical difference between the other two groups. A large number of studies have shown that 25OHD has a protective effect on islet β cell function and islet autoimmune and inflammatory responses [23]. Vitamin D deficiency has been linked to an increased incidence of T1DM worldwide, and new research suggests that vitamin D is associated with the incidence of DKA. In a study of 185 children with T1DM in the United States, 33% of the patients had DKA. Out of these cases, the incidence of DKA was 44% in the low vitamin D group and 18% in the vitamin D adequate group: the incidence of DKA in the vitamin D deficient group was significantly higher than that in the vitamin D adequate group [24]. However, an Australian study of the relationship between 25OHD and acidosis showed that acidosis may lead to impaired 1-α-hydroxylase activity to affect the metabolism of vitamin D, or low levels of vitamin D may be T1DM children’s major risk factors of DKA, remains to be further discussed [25]. Logistic regression analysis in this study showed that 25OHD appeared to be an independent factor for DKA, which is consistent with the findings of other studies.

To our knowledge, Henan Province is the third most populous province in China, and our research class reflected the onset characteristics of children with type 1 diabetes in China. This study specifically described the demographic and economic characteristics of hospitalization for new-onset diabetes in children, but is a single-center retrospective study. In the future, we will improve the multicenter study to further describe the clinical characteristics of new-onset diabetes in children in different regions of China. Our study only included limited clinical and laboratory indicators such as C-peptide and vitamin D. In future studies, the effects of children’s growth and development indicators, diet structure, lifestyle, genetics, ZnT8 antibody, etc. on the pathogenesis of diabetes will be further described.

## Conclusions

The incidence of DKA in children and adolescents aged 0–18 years in our province is high. Moreover, the diagnosis level of T1DM in our province needs to be improved. Younger children with T1DM are more likely to have DKA, delayed diagnosis, and more serious metabolic disorders. This leads to longer hospital stays and higher medical costs.

## Data Availability

Due to our need for subsequent studies, the datasets generated and/or analyzed in the current study are not publicly available, but are available at the reasonable request of the corresponding author.

## Abbreviations

25OHD: 25-hydroxyvitamin D
DKA: Diabetic ketoacidosis
GADA: Glutamate decarboxylase antibody
HbA1c: Hemoglobin A1C
IAA: Insulin antibody
ICA: Islet cell antibody
ISPAD: International Society of Pediatric and Adolescent Diabetes
T1DM: Type 1 diabetes mellitus
WHO: World Health Organization
